# The medial prefrontal cortex is crucial for the maintenance of persistent licking and the expression of incentive contrast

**DOI:** 10.3389/fnint.2015.00023

**Published:** 2015-03-27

**Authors:** Marc A. Parent, Linda M. Amarante, Benjamine Liu, Damian Weikum, Mark Laubach

**Affiliations:** ^1^The John B. Pierce Laboratory, New Haven, CTUSA; ^2^Department of Neurobiology, Yale School of Medicine, New Haven, CTUSA; ^3^Department of Biology and Center for Behavioral Neuroscience, American UniversityWashington, DC, USA; ^4^Yale University, New Haven, CTUSA

**Keywords:** reward processing, sucrose, learning, prefrontal cortex, cingulate cortex, muscimol, optogenetics

## Abstract

We examined the role of the medial prefrontal cortex (mPFC) in reward processing and the control of consummatory behavior. Rats were trained in an operant licking procedure in which they received alternating access to solutions with relatively high and low levels of sucrose (20 and 4%, w/v). Each level of sucrose was available for fixed intervals of 30 s over 30 min test sessions. Over several days of training, rats came to lick persistently when the high level of sucrose was available and suppressed licking when the low level of sucrose was available. Pharmacological inactivations of the mPFC, specifically the rostral part of the prelimbic area, greatly reduced intake of the higher value fluid and only slightly increased intake of the lower value fluid. In addition, the inactivations altered within-session patterns and microstructural measures of licking. Rats licked equally for the high and low levels of sucrose at the beginning of the test sessions and “relearned” to reduce intake of the low value fluid over the test sessions. Durations of licking bouts (clusters of licks with inter-lick intervals <0.5 s) were reduced for the high value fluid and there were many more brief licking bouts (<1 s) when the low value fluid was available. These effects were verified using an alternative approach (optogenetic silencing using archaerhodopsin) and were distinct from inactivation of the ventral striatum, which simply increased overall intake. Our findings suggest that the mPFC is crucial for the maintenance of persistent licking and the expression of learned feeding strategies.

## Introduction

The prefrontal cortex is crucial for using learned rules to control behavior (Miller and Cohen, 2001). Lesion studies in rodents have established that rule-guided behavior depends on the prelimbic cortex (aka area 32), which is part of the medial prefrontal cortex (mPFC; Delatour and Gisquet-Verrier, 1996, 1999). This cortical region is found in all mammals (Laubach, 2011) and is thought to have homologies across species both anatomically (Vogt et al., 2013; Vogt and Paxinos, 2014) and functionally (Narayanan et al., 2013). Damage in this mPFC region alters response times to external stimuli and increases the temporal variability of action (Stuss et al., 2003, 2005; Narayanan et al., 2006; Horst and Laubach, 2009). Such effects are not found following inactivation of the adjacent premotor cortex (Smith et al., 2010). Inactivation of the mPFC also alters the pace at which a given behavioral task is performed (Horst and Laubach, 2009). These effects may be due to the role of the mPFC in behavioral inhibition, as a number of studies have found that the mPFC is involved in controlling actions that must be postponed over extended periods of time, i.e., the ability to wait before acting (Muir et al., 1996; Passetti et al., 2002; Chudasama et al., 2003; Risterucci et al., 2003; Narayanan et al., 2006; Bari and Robbins, 2013). Another key function of the mPFC is adaptive control, the process through which behavioral outcomes are monitored and used to guide future behavior. In tasks that require inhibitory control, inactivation of the mPFC impairs measures of adaptive control such as post-error slowing in reaction-time tasks (Narayanan and Laubach, 2008; Narayanan et al., 2013). Neural recording studies have established that mPFC neurons track the execution of actions over time (Narayanan and Laubach, 2006, 2009) and encode current and past behavioral outcomes (Narayanan and Laubach, 2006, 2008; Totah et al., 2009). An encoding of behavioral outcome may arise through modulations of mPFC neurons that are phase locked to the onset of reward consumption (Horst and Laubach, 2012, 2013).

The studies reviewed above focused on the role of the mPFC in the control of complex operant behaviors. To our knowledge, no study has selectively disrupted mPFC processing during purely consummatory behaviors, which activate many mPFC neurons (Horst and Laubach, 2013). To this end, we developed a novel operant procedure based on classic studies of reward contrast (Flaherty, 1982). Rats received fluid for licking on a spout and the concentration of sucrose switched between a high level (20%) and low level (4%) every 30 s. Rats learned to lick persistently when the higher level of sucrose was available and to suppress licking when the lower value fluid was available. Based on the established role of the mPFC in behavioral inhibition, we expected to find that inactivation of the mPFC would increase consumption of the lower value fluid. Instead, mPFC inactivation reduced intake of the higher value fluid and reduced the duration of licking bouts. These effects were specific to the most rostral part of the mPFC, which selectively projects to feeding-related regions of the lateral hypothalamus and periaqueductal gray (Floyd et al., 2000, 2001; Gabbott et al., 2005) and is extensively interconnected with the agranular insular cortex (Gabbott et al., 2003) that contains neurons which respond selectively to gustatory (Yamamoto et al., 1989) and visceral (Cechetto and Saper, 1987; Allen et al., 1991) stimuli. To determine if these effects were specific to the mPFC, another cohort of rats was tested with inactivation of the ventral striatum, which simply increased overall intake similar to previous studies (Basso and Kelley, 1999; Eagle et al., 1999; Reynolds and Berridge, 2001; Baldo et al., 2005). Together, our studies suggest that the mPFC is necessary for the maintenance of persistent licking and the expression of learned feeding strategies.

## Materials and Methods

All experimental procedures were approved by the Animal Care and Use Committee at The John B. Pierce Laboratory, where the experimental studies were carried out, and conform to guidelines for the Ethical Treatment of Animals (National Institutes of Health).

### Subjects

Male Sprague-Dawley and Long Evans rats weighing between 350 and 375 g were purchased from Harlan Animals and were allowed 1 week to acclimate to their new environment with daily handling prior to training. Food access was regulated for maintenance of 90% of their free access body weight by giving 18 g of rat chow a day in the evenings following experiments. Rats weighed between 325 and 500 g at the time of testing. A total of 20 rats were tested for effects of positive contrast and 24 rats were tested for effects of negative contrast. Pharmacological inactivations were performed during the operant task in 27 rats and six of these rats were removed from the study due to improper cannula targeting. Seven rats (not included in the operant study) were tested with pharmacological inactivation of the mPFC during sucrose consumption in home cage. A total of 12 rats were used for optogenetic inactivation experiments and seven of these rats were removed from the study due to lack of viral expression and/or misalignment of the optic ferrule with the viral expression field.

### Behavioral Apparatus

All animals were trained in operant chambers containing a house lamp and enclosed within a sound-attenuating external chamber (ENV-008; Med Associates). Control of pumps and data acquisition was carried out using Med-PC version IV (Med Associates). A custom-made, multi-line drinking spout (John B. Pierce Laboratory Instruments Shop) was used that permitted multiple solution lines to merge and be consumed at a single point. The spout was located on one wall of the chamber at a height of 6.5 cm from the chamber floor and positioned horizontally. The spout was positioned between two barriers that restricted the movement of animals while licking on the spout. Two solution lines connected to 60ml syringes were used in these experiments. Syringe plungers were driven by single speed, syringe pumps (PHM-100; Med Associates) triggered by licks and delivered ~0.025 ml of fluid per activation. Licks were detected and recorded optically via interruptions of an infrared beam between an emitter and detector placed directly in front of the licking spout.

### Operant Licking Task

Animals were placed within an operant box for 30 min sessions with continual access to the drinking spout. Standard sessions consisted of shifts in the availability of the high value sucrose solution (20% sucrose wt/v) and low value sucrose solution (4% sucrose wt/v) over alternating thirty second epochs. Licking on the spout triggered delivery of the solutions. At the end of 30 s, the current epoch would end and the alternate solution epoch would commence following the next lick. Alternation between epochs continued until the 30 min session terminated.

Rats were tested for effects of positive and negative contrast in special sessions in which only the high or low value sucrose solution was provided. Positive contrast was detected as an increase in the consumption of the high value sucrose in sessions with access to both the high and low values of sucrose compared to sessions with access only to the high value sucrose. Negative contrast was detected as a reduction in the consumption of the low value sucrose in sessions with access to both the high and low values of sucrose compared to sessions with access to only the low value. We compared lick counts and measures of licking microstructure (number of bouts, bout duration, inter-bout interval) during the 30 s epochs when rats would normally receive the high or low value sucrose and by averaging these measures across all task epochs. In addition, we checked for potential changes in satiation using cumulative record plots of licking in each testing session.

### Surgeries

Animals were provided 2–3 days of full food access prior to implantation of either guide cannulas or infusion of virus into mPFC followed by implantation of optic ferrules. Animals were initially anesthetized with isoflurane (3.5%, ~2 min) and injected intraperitoneally with ketamine (100 mg/kg) and xylazine (10 mg/kg) for deep anesthesia throughout the surgery. When necessary, supplemental doses of ketamine and xylazine were administered over the span of the surgeries. The scalp was shaved and carprofen (10 mg/kg; Pfizer) was injected subcutaneously into the skin of the neck. Animals were placed into a stereotaxic apparatus using ear bars, the eyes covered in ophthalmic ointment, and the scalp was covered in iodine for 1 min. Iodine solution was wiped off the scalp and 0.3 ml of 2% lidocaine solution was injected subcutaneously along the midline. A midsagittal incision through the skin covering the scalp was made to expose the surface of the skull and the positioning of the head within the stereotaxic apparatus was adjusted to ensure lambda and bregma were leveled into the same plane. Four skull screws were placed caudally into the parietal skull bone plates for anchoring of implants to the head. Single 26 gage guide cannulas with dummy cannulas (Plastics One) were implanted bilaterally into the prelimbic region of the mPFC [anteroposterior (AP), +3.6; mediolateral (ML), +1.4; dorsoventral (DV), -4.0 from the surface of the brain at an angle of 12° from the midline]. Four rats had bilaterial dual cannula targeting both rostral and caudal levels of the mPFC (AP, +3.4; ML,±1.4; DV, -4.0 from the surface of the brain at an angle of 12° from the midline; this is the midpoint between the dual cannulas having a distance of 0.5 mm between the centers of each cannula). Cannulas targeted to ventral striatum were also implanted bilaterally (AP, +1.1; ML, ±4.3; DV, -5.5 from the surface of the brain at an angle of 22° from the midline). Guide cannulas were positioned 1 mm dorsal from the target brain structure due to using injection cannulas that extended 1 mm beyond the tip of the guide cannula.

For optogenetic experiments, methods were as above. Adeno-associated virus (AAV; serotype 9) plasmids containing archaerhodopsin (ArchT) and GFP driven under the ubiquitous CAG promoter (AAV-CAG-ArchT-GFP) were purchased from the University of North Carolina Vector Core (Chapel Hill, NC, USA). Viral titer was 2 × 10^12^ vm/ml. Implanted optic ferrules (Prizmatix) had an optic fiber with a diameter of 400 μm and a numerical aperture (NA) of ~0.66 with a flat cut tip to focus light delivery to regions directly below the end of the ferrule. 33 gage injection cannulas targeted only the rostral prelimbic cortex and 1 μl of virus was deposited at 0.1 μl/min using the same method described for infusion of drugs into the brain. Following injection of virus, injection cannulas were left in place for an additional 5 min to allow for diffusion of the virus into the brain prior to being retracted. Optic fibers were positioned 0.3 mm dorsal to the point of viral infusion.

After implantation of cannulas or ferrules, the craniotomy was covered and cannulas initially secured using cyanoacrylate (Slo-Zap) and cyanoacrylate accelerator (Zip Kicker). The entire implant was then permanently affixed to the skull by methyl methacrylate dental cement (AM Systems) via the four implanted skull screws. The skin surrounding the implants was cleaned, stapled taught around the implants, and covered in antibiotic ointment. Immediately following surgery, animals were given the α_2_-adrenoceptor antagonist yohimbine (2 mg/ml at equal volumes of the xylazine used during the surgery) to counteract the xylazine used for chronic anesthesia. To reduce any chances of infection the antibiotic enrofloxacin (23 mg/kg; Bayer) was given intraperitoneally when the animal began to move. Further, enrofloxacin was administered via the drinking water (70 mg/500 ml) throughout recovery. Carprofen was also administered via the drinking water (25 mg/500 ml) for 2 days following surgery for pain management. To prevent animals from pulling out dummy cannulas over the span of recovery and re-initiation of behavioral experiments, Kwick-Cast silicon sealant (WPI) was spread over the dummy cannula caps to secure them to their guide cannulas. Animals were allowed to recover from surgery for 1 week while having free access to food and water. Animals were monitored and weighed daily throughout recovery. Regulated access to food was reinstated several days prior to behavioral testing.

### Pharmacological Inactivations

Following training, recovery from surgery, and 4 days of re-exposure to the contrast task, animals underwent a series of control sessions prior to experimental infusion of drugs. First, a gas control where animals were exposed to the same light-level of isoflurane used during the experimental drug infusions (9–12 min @ 1.75%) and allowed to recover for 1 h prior to running the contrast task. Second, animals were tested after infusions of sterile PBS. In these sessions, animals were lightly anesthetized with isoflurane and targeted brain areas were infused with the PBS vector used to deliver muscimol. Following these control experiments, animals were lightly anesthetized with isoflurane and muscimol (1 μg/μl; Tocris or Sigma) or fluorescent conjugated muscimol (FCM; 5 μg/μl; BODIPY TMR-X conjugate; Life Technologies, as first used in Narayanan et al., 2006; see Allen et al., 2008 for methodological details) was delivered. Finally, the animals were given recovery sessions. All animals recovered within one or two test sessions after inactivation and showed equivalent intake and licking behavior to the PBS control sessions.

Drug delivery was carried out via a 33 gage injection cannula (Plastics One) connected to fine bore (0.38 ID) polythene tubing, filled with mineral oil that was lightly colored pink with 0.5% Oil RedO (Sigma; O0625). The tubing was connected to a 10-μl Hamilton syringe (Hamilton), also filled with mineral oil, and the injection needle was then back filled with drug. Drug was injected at 0.25 μl/min using a microsyringe pump (KD Scientific) and confirmed visually by marking the interface between the pink mineral oil and the infused solution before and after activation of the pumps.

### Optogenetic Inactivations

Green light (~520 nm) was provided continuously over 30 s epochs by a high power (100 mW) LED system (Prizmatix) controlled via TTL outputs from the Med PC system. A single fiber optic cable from the LED was connected to the input of an optic rotary joint (Prizmatix). The output of the rotary joint was then connected to a splitter optic cable for providing light to bilaterally implanted optic ferrules (Prizmatix). Up to ~18 mW of green light could be emitted at the tip of ferrule, as measured using an integrating sphere photodiode power sensor (Thor Labs; S140C) connected to a digital laser power and energy meter console (Thor Labs; PM100D). However, experiments were carried out with ~10 mW power at the tip of the fiber to limit the potential impact of heating the brain. The estimated irradiance at a distance of 0.3 mm (the distance from the center of the injection site to the tip of the optic fiber, as stated in the section above) was calculated as 16.36 mW/mm^2^, using the brain tissue light transmission calculator provided by the Deisseroth laboratory^1^.

### Verification of Cannula Placement, FCM, Viral Expression, and Optic Ferrule Placement

Following experimentation, animals were initially sedated with isoflurane (3.5% for 3 mins) and injected intraperitoneally with 1ml of Euthasol. The animals were transcardially perfused first with 200 ml cold saline solution followed by 200 ml of cold 4% paraformaldehyde. Brains were extracted and placed into 4% paraformaldehyde containing 20% sucrose and 20% glycerol. Brains were cut to 100 μm thick slices in the frontal plane. For experiments using fluorescent material, half of the acquired slices were mounted to slides using Vectashield fluorescent mounting medium containing DAPI (Vector Labs) and the other half were Nissl stained via treatment with thionin. Alternatively, only Nissl stained slices were mounted to slides from brain not using some form of fluorescent material. Imaging of slices was done on a Motic BA400 microscope, QImaging digital camera, and ASI MS2000 motorized stage. Visualization of fluorescence material in brains was provided for by a Photofluor fluorescent light source using DAPI, TRITC (rhodamine), and FITC filters (Motic). Images were acquired using Bioquant V8.40.20 software.

### Behavioral Data Analysis

All data were analyzed using custom-built scripts in Matlab version R2012b (Mathworks) and statistics were performed in R x64 ver. 2.15.1 using R-Studio ver. 0.97.237^2^. Bouts of licks were detected using custom Matlab code provided by Dr. Ranier Gutierrez, as used previously in Gutierrez et al. (2006) and Horst and Laubach (2013). Specifically, bouts were defined as having at least three licks within 300 ms and with an inter-bout interval of 0.5 s or longer. Lick counts and bout durations were significantly correlated (*r* = 0.835) over all rats tested.

### Sucrose Consumption in Home Cage

Seven rats, used in other drug infusion studies that are not reported in the present manuscript, were presented with calibrated drinking tubes containing 20 and 4% sucrose (w/v) for a period of 5 min in their home cages. Volumes consumed were noted over a series of 9 days. On the 10th day, rats were tested 1 h after a control infusion procedure (light anesthesia via isoflurane over a period of ~12 min). On the 11th day, they were tested 1 h after an infusion of muscimol (as in the operant studies above). One rat showed a lack of evidence for drug infusion and another rat did not consume any fluid during the control or muscimol test sessions. These animals were removed from the data summaries for home-cage testing reported below.

## Results

### Positive and Negative Reward Contrast Effects in the Operant Licking Task

**FIGURE 1:**
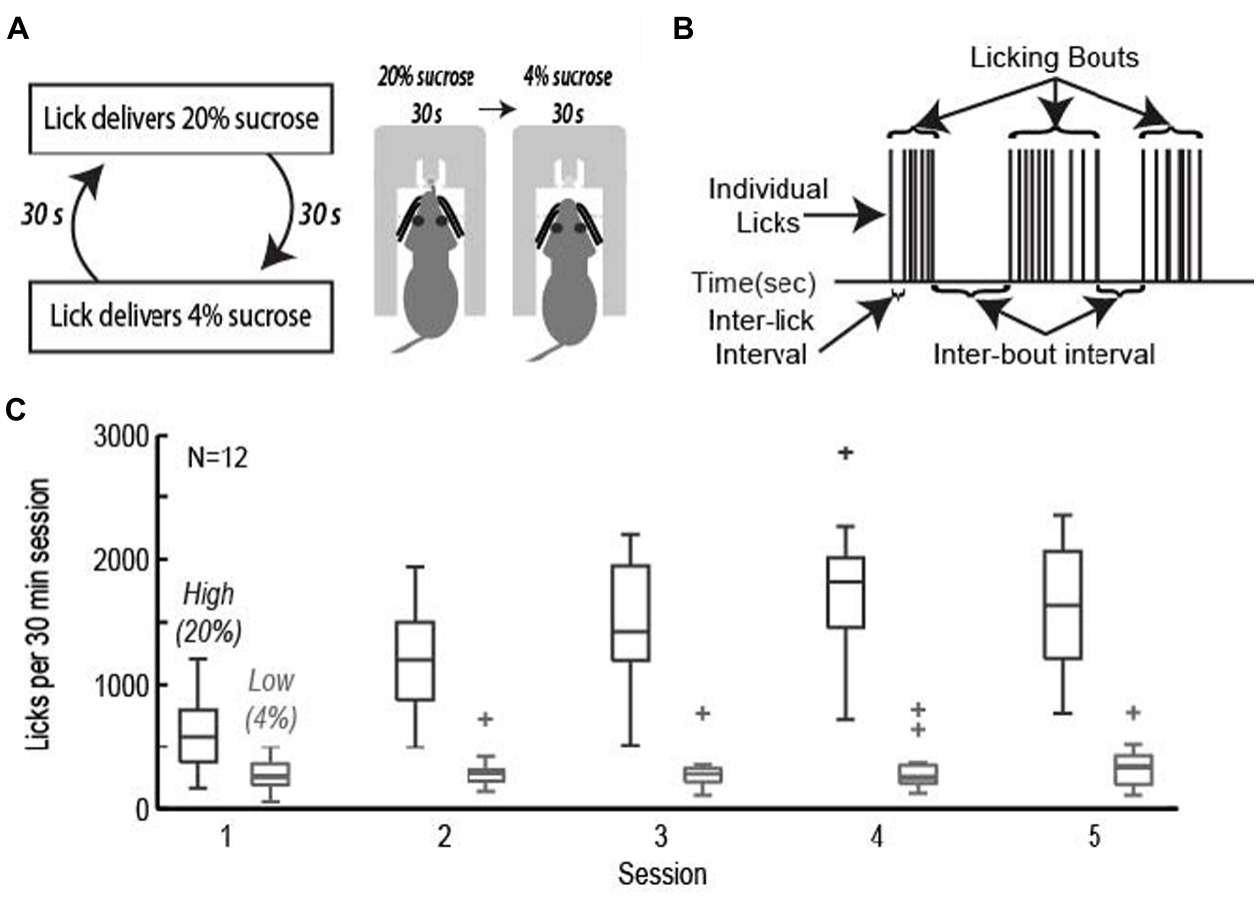
**The operant licking task. (A)** Rats licked on a single spout and received access to solutions containing relatively high (20%, w/v) or low (4%) sucrose. The two fluids were presented in alternating 30-s epochs at the same spout. **(B)** Animals consumed sucrose by emitting bouts of licks. Key measures of licking “microstructure” are indicated. **(C)** Acquisition of learning. Rats learned to actively suppress licking for the low value (4%) sucrose solution while gradually increasing intake for the high value (20%) sucrose solution across the first five sessions of the task.

Rats were trained to perform an operant licking task in which they received access to fluids containing relatively high and low levels of sucrose over alternating 30-s epochs (**Figure 1A**). Fluids were delivered from a single common spout containing two inner tubes connected to tubing and two independently driven peristaltic pumps. Intake of liquid foods via licking has been characterized extensively using a “microstructural analysis” of licking (Davis and Smith, 1992; Davis and Perez, 1993; Grigson et al., 1993; Davis, 1996; Spector and St John, 1998; Spector et al., 1998; Kaplan et al., 2001). These methods were used in the present study. **Figure 1B** demonstrates that licking did not occur with a constant rate. Instead, licking largely occurred in bursts, termed licking bouts, having a stable (6–7 Hz) intra-bout lick rate that is independent of the relative sucrose concentration. Modulation of intake was implemented through adjustments in bout duration, number of bouts, and ultimately total number of licks.

Rats achieved asymptotic levels of licking over four to seven daily training sessions. They came to lick persistently when 20% sucrose was available and emitted many fewer licks when 4% sucrose was available (**Figure 1C**). A two-factor repeated measures ANOVA with factors for training session and sucrose concentration found that there was a significant change in mean lick count per epoch [*F*(3,238) = 27.87, *p* < 0.001], total number of licks per session [*F*(3,238) = 34.13, *p* < 0.001], number of bouts [*F*(3,238) = 8.971, *p* < 0.001], and duration of bouts [*F*(3,236) = 6.485, *p* < 0.001]. The significant interaction between training session and sucrose concentration led us to perform single-factor ANOVAs on the measures of licking for each of the sucrose levels. Intake of the low value solution was relatively stable and did not decrease significantly over days [mean licks per epoch: *F*(3,136) = 1.33, *p* > 0.3; total number of licks per session: *F*(3,136) = 0.45, *p* > 0.7; number of bouts: *F*(3,136) = 1.37, *p* > 0.2; duration of licking bouts: *F*(3,136) = 1.71, *p* > 0.1]. There was a significant increase in mean licks per epoch [*F*(3,136) = 23.36, *p* < 0.001], total number of licks per session [*F*(3,136) = 26.35, *p* < 0.001], number of bouts [*F*(3,135) = 13.94, *p* < 0.001], and duration of licking bouts [*F*(3,135) = 6.609, *p* < 0.001] when the higher value solution was available.

**FIGURE 2:**
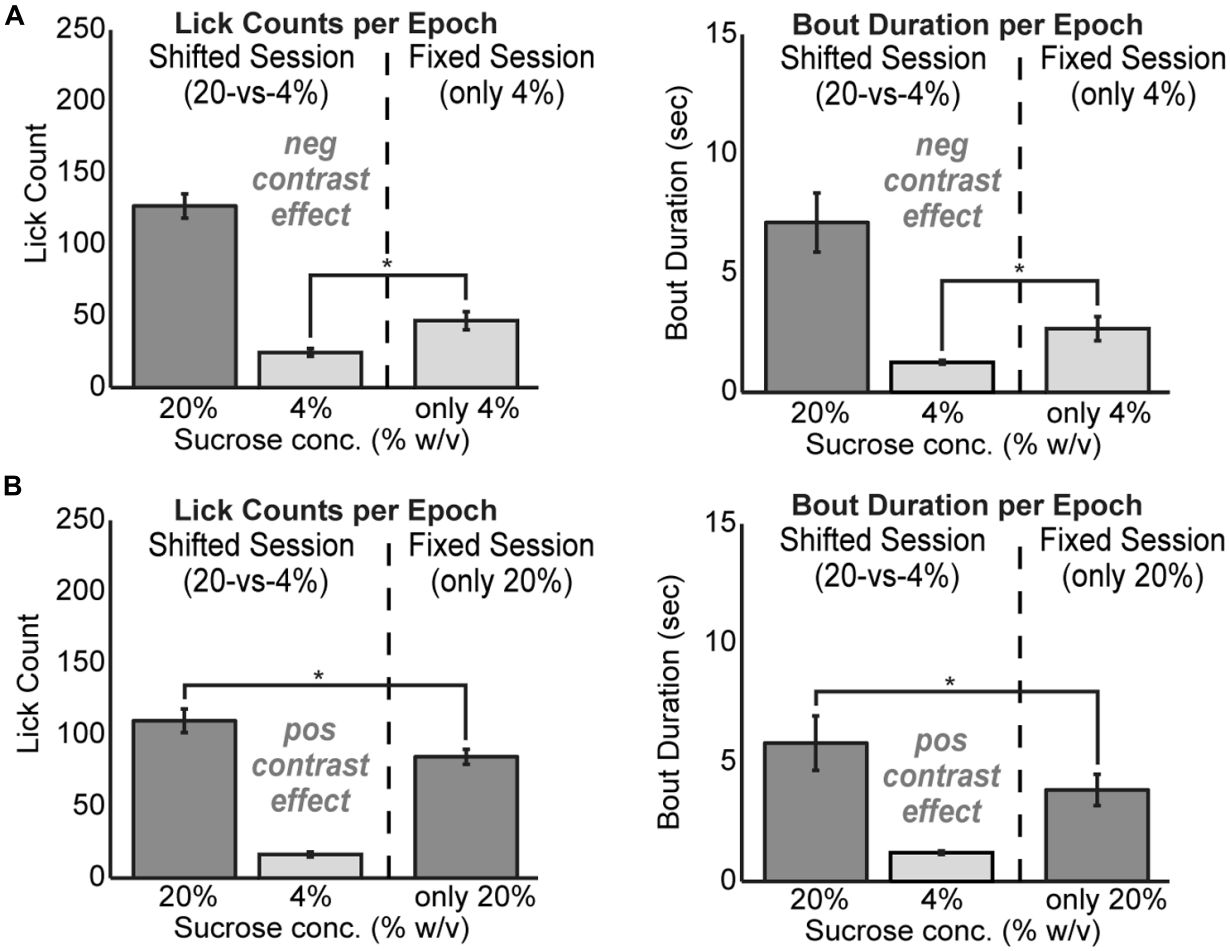
**Reward contrast effects were revealed by testing rats with only one level of sucrose in a given behavioral session**. Contrast effects were apparent both in terms of lick counts and the average duration of licking bouts. **(A)** Negative contrast was evident as rats (*N* = 24) consumed more of the low value solution when it was presented alone compared to the standard sessions with alternating access to the two levels of sucrose. **(B)** Positive contrast was evident as rats (*N* = 20) consumed less of the high value solution when it was presented alone compared to the sessions with alternating access to the two levels of sucrose.

To test for positive and negative contrast effects in the task, rats were tested with fixed levels of sucrose over the entire 30 min behavioral session. Rats demonstrated clear evidence for both forms of contrast. Negative contrast effects were found in testing of 24 rats. The mean lick count over epochs [*F*(1,23) = 20.01, *p* < 0.001] and duration of licking bouts [*F*(1,23) = 7.706, *p* < 0.05] were significantly lower during consumption of the low value fluid when it was presented in alternating series with the higher value fluid compared to when only the low value fluid was available (**Figure 2A**). Conversely, positive contrast effects were found in testing of 20 rats. The mean lick count over epochs [*F*(1,19) = 18.22, *p* < 0.001] and duration of licking bouts [*F*(1,19) = 8.121, *p* < 0.05] were significantly higher during consumption of the high value fluid when it was presented in alternation with the low value fluid compared to when only the high value fluid was available (**Figure 2B**).

### Inactivation of mPFC Reduces Consumption and Temporally Fragments Licking

**FIGURE 3:**
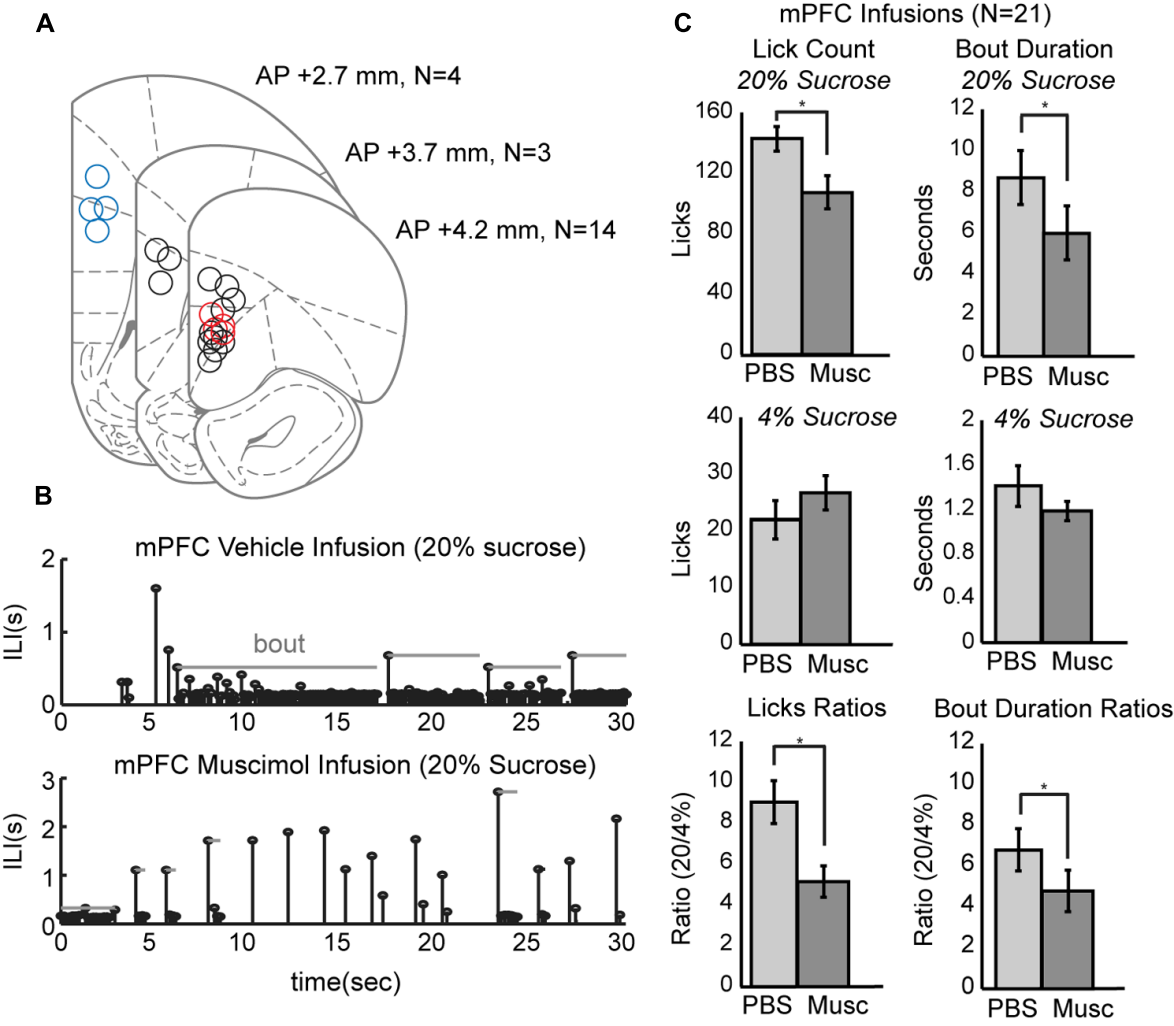
**Effects of reversibly inactivating the mPFC in the operant licking task. (A)** Coronal depiction of infusion sites for muscimol within the mPFC. Sites at 3.7 mm A/P were considered to be in the rostral mPFC area. Nine of these sites were clearly within the prelimbic area. Sites at 2.7 mm were considered to be in the caudal mPFC area. Dual cannula located in both rostral (red) and caudal (blue) mPFC are included as well. **(B)** Temporal patterns of licking were dramatically altered by inactivation of mPFC. Top: Licking for 20% sucrose following infusion of PBS vehicle occurred in sustained bouts. Bottom: Licking following inactivation of mPFC with muscimol (Musc) was associated with reduced bout durations and reduced overall licking. **(C)** Inactivation of mPFC decreased licking and the duration of licking bouts for the higher, but not the lower, value fluid and reduced the ratios of licking for the higher-to-lower value fluids (a measure of incentive contrast).

To study the role of mPFC in the regulation of intake, pharmacological methods for reversible inactivation were used to inactivate the mPFC during the operant licking task. Twenty-one infusions of 1 μl muscimol (1 μg/μl) or fluorescent conjugated muscimol (5 μg/μl) were made in 17 rats. **Figure 3A** depicts the estimated placement of all mPFC cannulas. Fourteen of the rats had bilateral cannulas located in the rostral mPFC (+4.2 mm AP). Nine of these rats had both cannulas clearly centered in the prelimbic cortex (area 32). Four of these rats also received a second pair of cannulas in the more caudal mPFC (+2.7 mm AP). Three rats had single bilateral cannulas at an intermediate location (+3.7 mm AP).

Temporal patterns of licking were dramatically altered by inactivation of mPFC (**Figure 3B**). This plot shows inter-lick intervals (ILIs) over experimental time during a 30-s epoch when the high-value sucrose was consumed by one of the rats in sessions with an infusion of PBS (top row) and muscimol (bottom row). Bouts of licks are denoted by the gray horizontal lines. Inactivation of the mPFC significantly decreased overall licking counts [paired *t*-test: *t*(20) = 4.204, *p* < 0.001] and decreased the duration of licking bouts [*t*(20) = 2.73, *p* < 0.05] for the higher-value, but not the lower-value, fluid (**Figure 3C**, top and middle rows).

Inactivation of mPFC also reduced the incentive contrast properties (aka subjective or relative value) of the liquid sucrose. To measure these effects, we calculated the ratios of lick counts and bout durations for when the high and low value sucrose was available. Inactivation of mPFC decreased these ratios for lick counts [*t*(20) = 3.87, *p* < 0.001] and bout durations [*t*(20) = 2.53, *p* < 0.05; **Figure 3C**, bottom row].

These effects cannot be explained by an impairment in sucrose intake due to mPFC inactivation, as a separate cohort of five rats were tested with mPFC inactivation during sucrose intake in the home cage. There was no effect of the inactivation on the volume of sucrose that was consumed. The rats drank 9.5 ± 3.3 ml of liquid sucrose in control sessions and 9.5 ± 1.9 ml in inactivation sessions.

### Effects of Inactivation on Measures of Incentive Contrast were Specific to the Rostral mPFC

**FIGURE 4:**
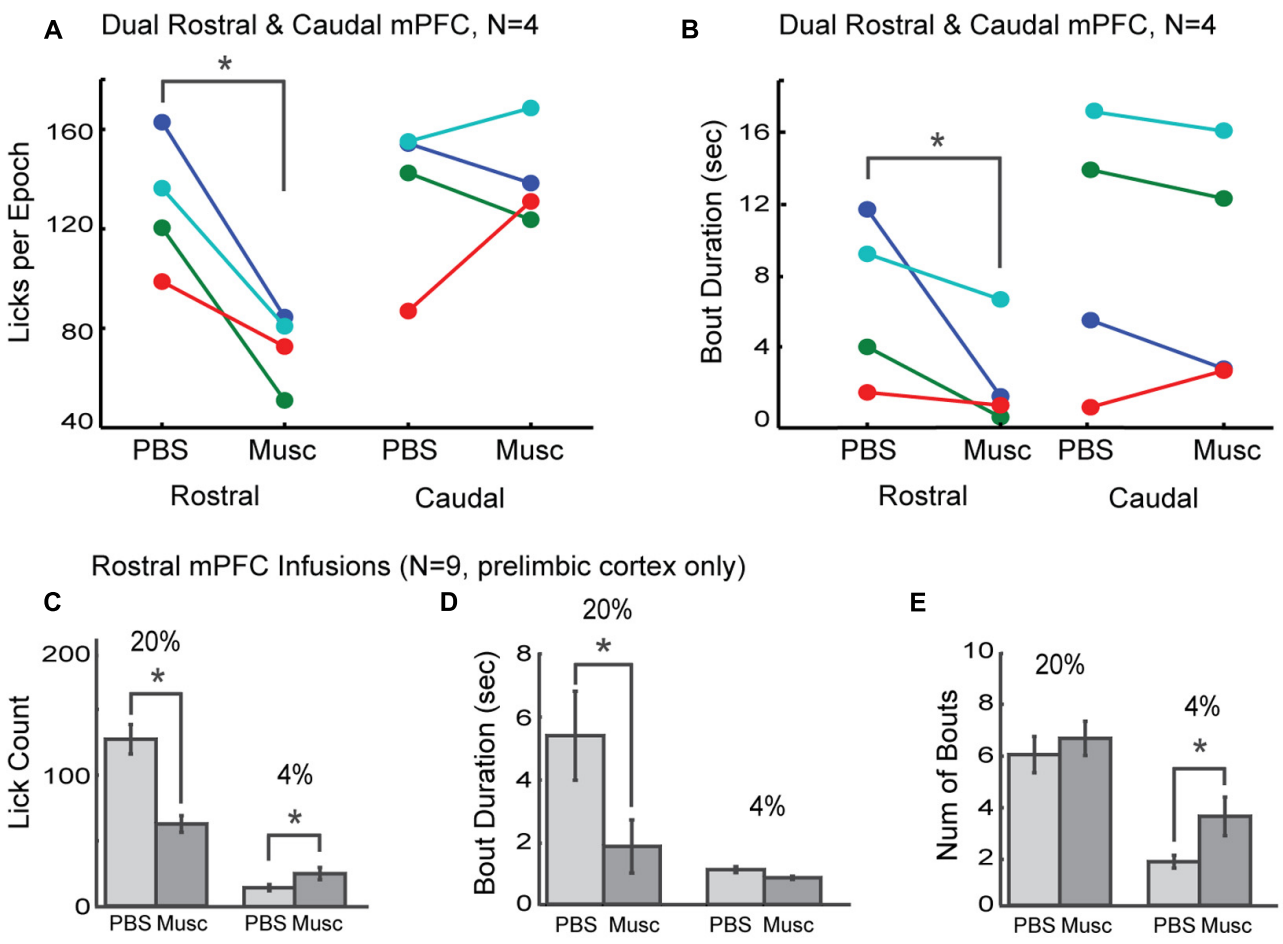
**Effects of inactivation on measures of incentive contrast were specific to the rostral mPFC. (A)** Inactivation of the rostral, but not the caudal, mPFC reduced licking for the higher value fluid. **(B)** Inactivations also reduced bout durations following rostral mPFC inactivation. No effects of caudal mPFC inactivation were found for any measure of licking microstructure, including bout duration. These data were collected in four rats with dual bilateral cannula (blue and red dots in **Figure 3A**). **(C)** Inactivation of rostral mPFC in nine rats (see cannula locations in **Figure 3A**) decreased licking for the high value (20%) sucrose and increased consumption of low value (4%) sucrose. **(D)** Bout duration for high, but not low, value sucrose was also reduced following inactivation. **(E)** There was no change in the number of licking bouts for the high value sucrose. However, more bouts were emitted when the low value fluid was available and the rostral mPFC was inactivated. *p < 0.05.

The prelimbic cortex exhibits differing hodology between its rostral and caudal divisions, with especially heavy projections from the rostral mPFC (~4 mm AP) targeting feeding-related regions in the lateral hypothalamus and periaqueductal gray (Floyd et al., 2000, 2001; Gabbott et al., 2003, 2005; Hoover and Vertes, 2007). These differences in connectivity may provide these brain regions with differential roles in the control of intake. To distinguish regional differences within the mPFC, dual bilateral cannulas were implanted in the rostral mPFC and caudal mPFC areas in four additional rats (colored markings in **Figure 3A**). Lick counts for the high-value fluid decreased by 51% [*t*(8) = -2.85, *p* < 0.05; **Figure 4A**]. Bout durations for the high-value fluid decreased by 66% [*t*(8) = -3.28, *p* < 0.05; **Figure 4B**]. These results suggest that inactivation of the rostral, but not the caudal, mPFC reduced persistent licking for sucrose.

As prior studies suggest that mPFC has a role in behavioral inhibition (Bari and Robbins, 2013), we hypothesized that inactivation of rostral mPFC would, in the least, disinhibit consumption of the low-value sucrose solution. This effect could be evident both as an increase in licking counts and the duration of licking bouts, especially for the lower-value 4% sucrose. To examine this issue, we compared several measures of licking for the high and low value sucrose for nine rats with their infusion cannulas localized to the rostral prelimbic cortex (see **Figure 3A** for cannula sites). Lick counts for the high-value fluid decreased by 51% [*t*(8) = -2.85, *p* < 0.05; left plots in **Figure 4C**] and bout durations for the high-value fluid decreased by 66% [*t*(8) = -3.28, *p* < 0.05; left plots in **Figure 4D**]. There was also a significant increase in licking when the low-value sucrose was available [*t*(8) = -2.85, *p* < 0.05; right plots in **Figure 4C**] but no change in bout duration [*t*(8) = 1.00, *p* > 0.05; right plots in **Figure 4D**]. The increase in lick counts for the lower value fluid was accompanied by an increase in the number of licking bouts for the low [*t*(8) = -2.64, *p* < 0.05], but not the high value fluid [*t*(8) = -0.55, *p* > 0.05; **Figure 4E**]. Together, these results establish that inactivation of the rostral mPFC did not alter inhibitory control over intake. Instead, the inactivations reduced persistent licking for the high-value fluid and increased the occurrence of brief licking bouts when the low-value solution was available. The changes in licking for the low-value solution might have been due to compensatory changes due to the inefficient licking (temporally fragmented bouts) that occurred with the rostral mPFC inactivated (**Figure 3B**). Indeed, the rats remained engaged in the task for a significantly longer duration before cessation of intake in the inactivation sessions [PBS: 1094 s; Muscimol: 1559 s; paired *t*-test: *t*(8) = 5.72, *p* < 0.05].

### Inactivation of the Rostral mPFC Increased the Variability of Licking

**FIGURE 5:**
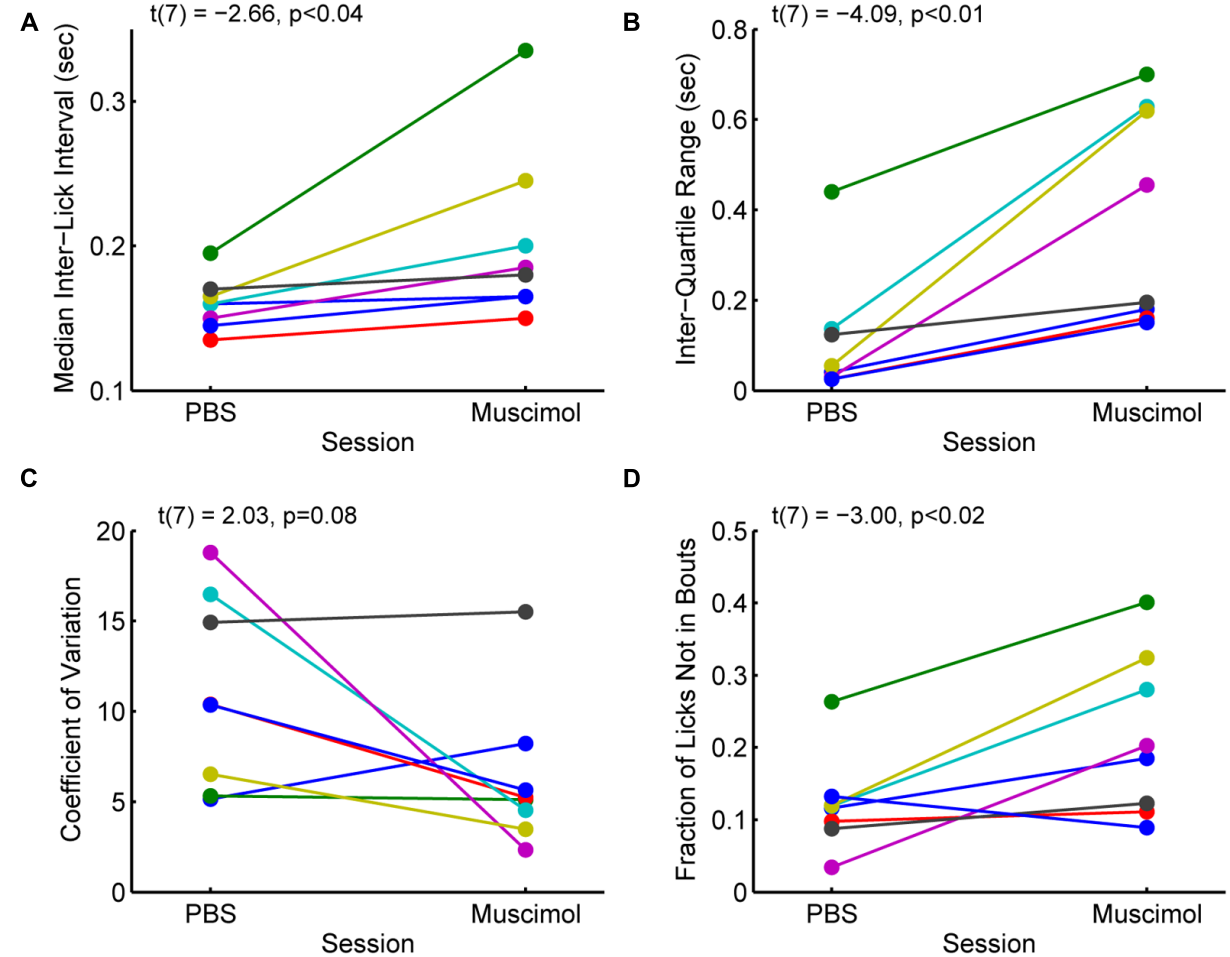
**Effects of inactivation on measures of licking variability. (A)** Inactivation of the rostral mPFC increased the median inter-lick interval [ILI; paired *t*-test: *t*(7) = -2.66, *p* < 0.04), defined as the time between pairs of licks, in 8 rats with muscimol infused into the rostral mPFC (see **Figure 3A**). **(B)** Inactivation also increased the inter-quartile range [*t*(7) = -4.09, *p* < 0.01], a non-parametric measure of variability, of the ILI. **(C)** There was no consistent effect of inactivation on another measure of variability, the coefficient of variance (*t*(7) = 2.03, *p* = 0.08). (Note that a coefficient of variance, defined as the variance divided by the mean, near 1 is typical for a random (Poisson) time series; **(D)** The fraction of non-bout licks (isolated licks and lick pairs) increased with inactivation [*t*(7) = -3.00, *p* < 0.02]. This finding is further support for the idea that the rats were less able to persistently lick with the rostral mPFC inactivated.

The changes in measures of incentive contrast (aka subjective value) described above were accompanied by increases in the variability of licking and increases in the fraction of non-bout licking (**Figure 5**). Eight rats with cannula implanted in the rostral mPFC (see **Figure 3A**) showed increases in the median ILIs [paired *t*-test: *t*(7) = -2.66, *p* < 0.04; **Figure 5A**] and the inter-quartile range for the ILIs [*t*(7) = -4.09, *p* < 0.01; **Figure 5B**]. That is, the rats licked more slowly and with more variability with the rostral mPFC inactivated. (A ninth rats included in the analysis reported in the section above and **Figure 4** was excluded from this analysis due to having much higher levels of variation (off the scale shown in **Figure 5** in its ILI.) No consistent effect was found on another measure of variability, the coefficient of variation [*t*(8) = 2.03, *p* = 0.08; **Figure 5C**), defined as variance divided by mean. This finding that suggests that while variability increased with rostral mPFC inactivation (based on the inter-quartile range of the ILIs), it did not result in a shift of licking to a totally random Poisson-like process (with a coefficient of variation near 1). However, there was a significant increase in the fraction of isolated licks and lick pairs [those licks that were not considered as bouts; *t*(8) = -3.00, *p* < 0.02; **Figure 5D**]. A simple interpretation of these findings is that inactivation of the mPFC altered sensorimotor aspects of licking, which may underlie the effects of inactivation on measures of incentive contrast.

### Optogenetic Perturbations Reproduced Effects of Pharmacological Inactivation

**FIGURE 6:**
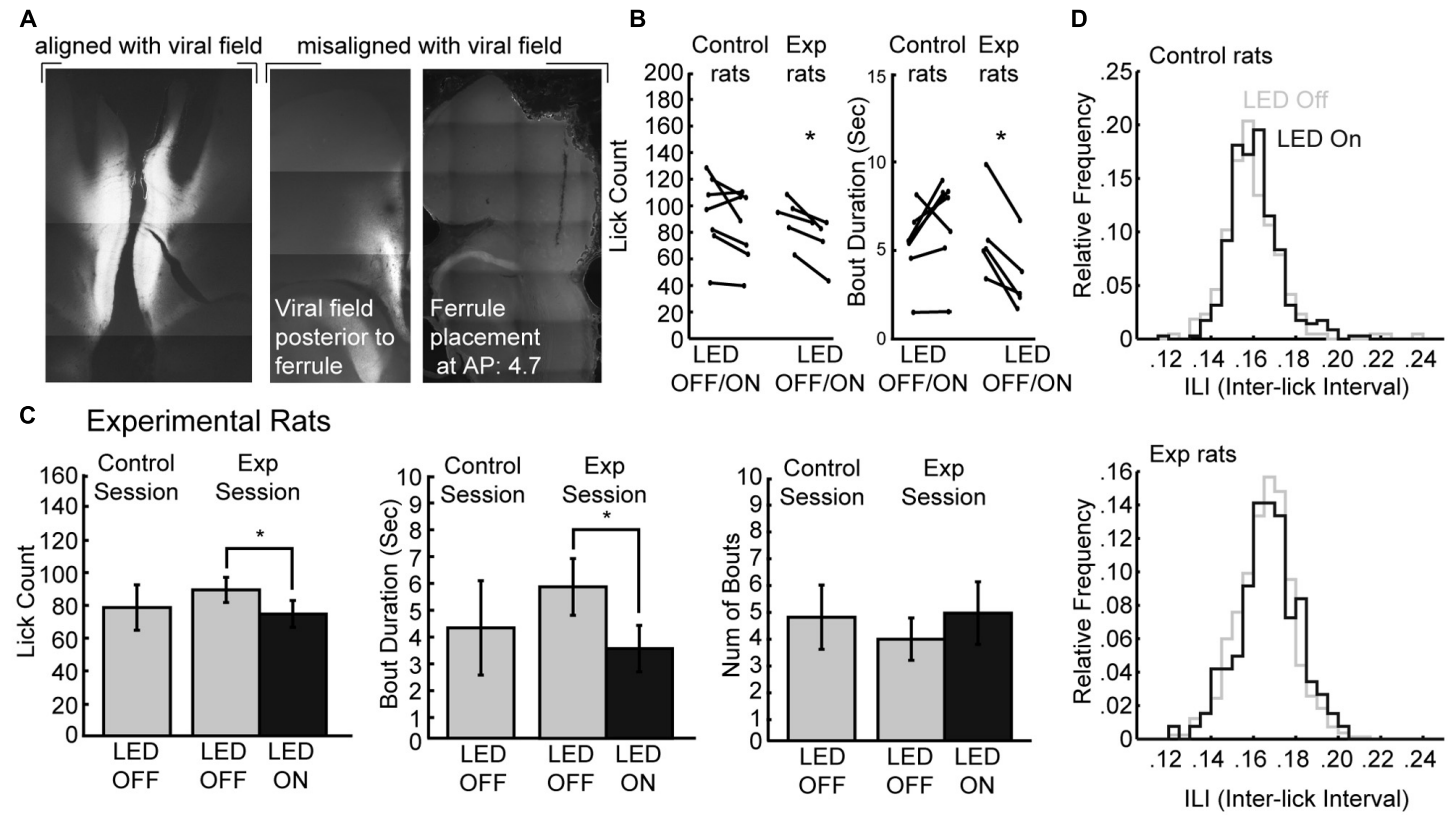
**Optogenetic perturbation of the rostral mPFC prevents persistent licking. (A)** Fluorescent GFP expression driven by the non-specific CAG promoter showed evidence for transduction of archaerhodopsin (ArchT) in the rostral mPFC of experimental rats. Control rats either did not show proper alignment of viral field with implanted ferrules or showed no viral expression. **(B)** Transient perturbation of the rostral mPFC in experimental rats via LED activation consistently decreased licking and bout duration. There was no consistent change in licking by LED in control rats. **(C)** Transient perturbation of the rostral mPFC in experimental rats via LED activation consistently decreased licking in epochs with LED on relative to epochs with LED off. The optogenetic perturbation of rostral mPFC in experimental rats also reduced the duration of licking bouts. There was no significant change in the number of bouts initiated with the LED activated. **(D)** The ability to lick within bouts was not affected as there was no significant change in the ILI histogram of licking during epochs between epochs with the LED on and the LED off. *p < 0.05.

The muscimol inactivation method alters brain processing for a period of several hours, e.g. (Allen et al., 2008) and it is not possible to demonstrate effects of inactivation within a given behavioral session. Recently developed optogenetic methods allow for within-session perturbations of neural activity. Here, we used archaerhodopsin (Han et al., 2011; Huff et al., 2013; Stefanik et al., 2013; Tsunematsu et al., 2013) to transiently silence neural activity during the operant licking task (**Figure 6**). Using this method, it was possible to perturb rostral mPFC-dependent licking in a manner similar to inactivation with muscimol. **Figure 6A** shows the extent of expression pattern of AVV-CAG-ArchT-GFP throughout the medial aspect of the frontal cortex when co-aligned with implanted ferrules, as found in the experimental group (*N* = 5), and when either misaligned (or non-expressing) in the control group (*N* = 7). The tips of the optic ferrules delivered green light (520 nm) from a 100 mW LED source. The LED system was calibrated to deliver ~10 mW from the tips of the bilaterally implanted optic fibers.

To determine if perturbations of rostral mPFC alters control of licking independent of incentive contrast effects, we tested the rats as they consumed a 20% sucrose solution. Alternating 30-s epochs were defined as either having the LED on or the LED off, and the LED was left on continuously for the entirety of the 30-s epoch. We found that in experimental rats there was a consistent decrease in both licking and the duration of licking bouts in all rats, but this was not the case in control rats (**Figure 6B**). A within-session comparison between epochs with the LED turned on and off revealed that there was significantly less licking during epochs of LED activation [paired *t*-test: *t*(4) = 4.387, *p* < 0.05] and bout durations were also significantly reduced [*t*(4) = 5.105, *p* < 0.01]. Mean lick counts and bout durations were reduced by roughly 17 and 40%, respectively (left and middle plots in **Figure 6C**). These changes in licking deviated from the means of the preceding day where both epochs had no LED operating (control sessions). There was no significant change in the number of bouts with ArchT activation (right plots in **Figure 6C**) nor ILI histograms (**Figure 6D**).

These results were obtained in five rats in which there was anatomical evidence (GFP fluorescence) for transduction of ArchT and alignment of the GFP marker for ArchT and the tips of the optic fibers. Seven rats had either a lack of GFP labeling or a mismatch between the GFP marker and the optic fibers. None of these rats showed any effects of LED activation on licking counts [*t*(6) = 1.658; *p* > 0.1] or bout duration [*t*(6) = 1.723; *p* > 0.1].

### Inactivation of the Rostral mPFC Altered within-Session Patterns of Licking

**FIGURE 7:**
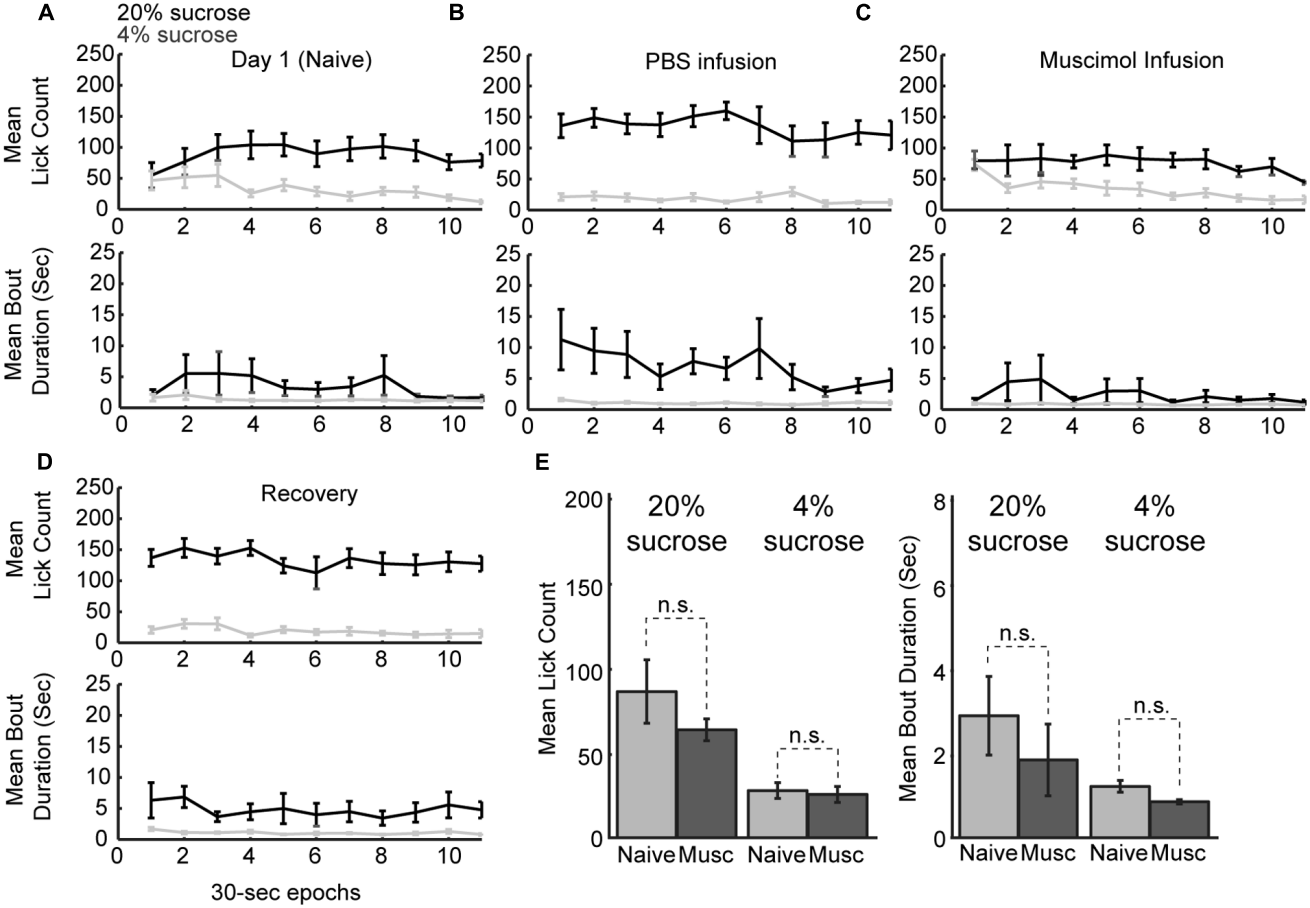
**Within-session dynamics following inactivation of the rostral mPFC. (A)** Epoch-by-epoch summary of mean lick counts and bout durations. Dark and gray lines represent access to the high- and low-value sucrose solutions, respectively. Naive animals begin consumption of high and low sucrose at the same level and inhibit consumption of low-value sucrose within the first day of training. **(B)** After training, sessions that followed PBS infusions showed high lick rates and long licking bouts during access to the high value solution and low lick rates with short bout durations during access to the low value solution. **(C)** Sessions that followed inactivation by muscimol were associated with equal initial intake of both the low and high value solutions and an eventual suppression of licking for the low-value fluid. This pattern of intake is comparable to consumption when animals are naïve in the first day of training. **(D)** In recovery sessions (day after inactivation), the normal patterns of licking reemerged. **(E)** Comparison of licking in the inactivation sessions and the first day of training (naïve animals) showed no significant difference in either lick counts or bout durations.

Naïve rats are unaware of the alternating access to high and low-value sucrose and required several days of testing to demonstrate incentive contrast like effects (**Figure 1C**). At the beginning of the testing sessions, they consumed both levels of sucrose with equal vigor and then showed increased consumption during high sucrose epochs and decreased consumption during low sucrose epochs (**Figure 7A**). Once trained, rats licked more for the high-value sucrose compared to the low-value sucrose throughout the behavioral test session (**Figure 7B**). Following inactivation of rostral mPFC, the rats returned to licking at equal rates for the two fluids at the beginning of the session and, similar to during the initial naïve session (**Figure 7C**). Licking was reduced over the inactivation sessions when the low-value fluid was available, a finding that suggests that the rostral mPFC inactivation did not eliminate the ability of the rats to suppress licking for the low-value fluid. These effects were not observed in recovery sessions that were run after the inactivation sessions (**Figure 7D**). Both lick counts and bout durations were reduced to levels found in the very first training sessions (**Figure 7E**). That is, there were no differences in licking between the first training session and the muscimol inactivation sessions in the well trained rats [20% sucrose (naïve vs. inactivated): Lick count: *t*(8) = 1.063, *p* > 0.05; Bout duration: *t*(8) = 1.606, *p* > 0.05; 4% sucrose (naïve vs. inactivated): Lick count: *t*(8) = 0.3464, *p* > 0.05; Bout duration: *t*(8) = 1.726, *p* > 0.05]. These findings suggest that inactivation of the rostral mPFC blocked the expression of the learned within-session licking patterns.

### Inactivation of Ventral Striatum Increased Overall Fluid Intake

**FIGURE 8:**
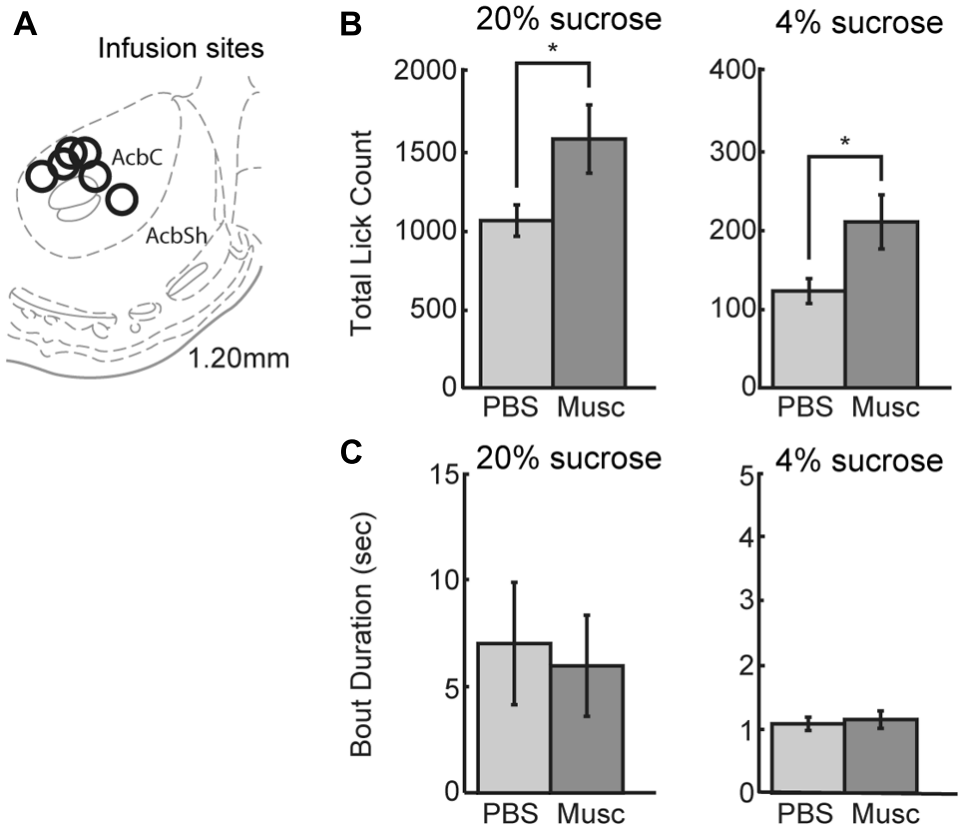
**Infusions of vehicle and muscimol were also performed in ventral striatum. (A)** Infusion sites were located in the nucleus accumbens core. **(B)** Opposing findings from mPFC, inactivations in ventral striatum led to an increase in lick count for the high value (20%) sucrose solution. The number of licks for the low value (4%) sucrose solution also increased during muscimol inactivation of ventral striatum. **(C)** There was no change in bout duration for the high or low value sucrose solution with muscimol inactivations of ventral striatum.

Previous studies (Eagle et al., 1999) suggest that inactivation of ventral striatum leads to increased overall intake. The ventral striatum, and not the mPFC, might be a source of inhibitory control over intake. To examine this issue using our operant incentive contrast task, six rats were implanted with drug infusion cannulas into nucleus accumbens (**Figure 8A**). (Given that muscimol is known to spread over an area of at least 1 mm^2^ (Martin and Ghez, 1999; Allen et al., 2008), we have characterized the affected area from these infusions as being in the ventral striatum and not the core of the nucleus accumbens.)

Inactivation of ventral striatum led to a significant increase in total licking over sessions for both the high and low levels of sucrose (**Figure 8B**). There was a 49% increase in the number of licks for the high-value sucrose [*t*(5) = 2.900, *p* < 0.04) and a 76% increase in the number of licks for the low-value sucrose [*t*(5) = 3.328, *p*< 0.03]. No significant effects were found for the microstructural measures of licking (**Figure 8C**). These findings suggest that the ventral striatum, and not the mPFC, has an inhibitory role in the control of sucrose consumption.

## Discussion

We examined the role of the mPFC in the control of consummatory behavior using an operant licking procedure (**Figure 1A**). Reversible inactivations of the rostral mPFC reduced the expression of incentive contrast (**Figure 3C**), temporally fragmented bouts of licking (**Figure 3B**), and increased several measures of licking variability (**Figure 5**). Common effects were obtained using the classic muscimol method and an optogenetic silencing method (**Figure 6**). Inactivation of the caudal mPFC had no significant effects on licking behavior (**Figure 4**) and inactivations of the ventral striatum (**Figure 8**) were distinct from those in the cortex. Inactivating the ventral striatum led to increased overall consumption especially for the lower value fluid. Together, these findings suggest that there is a regional specialization for the control of orolingual behavior within the mPFC (rostral, not caudal) and between the mPFC and ventral striatum.

Our operant licking procedure was designed based on classic studies of consummatory reward contrast (Flaherty, 1982). Testing with alternating access to solutions with relatively higher and lower concentrations of sucrose revealed positive and negative contrast effects (**Figure 2**). That is, the rats drank more of the higher valued fluid and less of the lower valued fluid when the fluids were presented in an interleaved manner compared to when only one of the solutions was available in the testing session. The presence of the two contrast effects allowed us to assess the potential roles of the mPFC in enhancing intake of the higher value fluid and inhibiting intake of the lower value fluid. Behavioral inhibition has been proposed to underlie the expression of negative contrast (Black, 1968; Lombardi and Flaherty, 1978; Flaherty, 1982) and is a core function of the mPFC (Bari and Robbins, 2013). Therefore, we expected to find that reversible inactivation of the mPFC would lead to a loss of self-control in the task, increasing overall intake and reducing negative contrast (i.e., increasing intake of the lower value fluid to the amount found in test sessions with only that fluid). Instead, we found that mPFC inactivation reduced intake of the higher value fluid (**Figure 3C**), similar to the loss of the positive contrast effect (**Figure 2B**), and only slightly increased licking for the lower-value fluid in the operant task. The inactivations increased the variability of licking (**Figure 5**), but did not reduce total licking in the operant task and did not reduce fluid consumption in testing sessions done in the home cage. These results suggest that the net effect of inactivating the mPFC on sucrose consumption in the incentive contrast task is not inhibitory.

An analysis of the within-session patterns of licking (**Figure 7**) adds further support to this interpretation. Rats normally began the testing sessions licking more for the higher level of sucrose and less for the lower level of sucrose. Inactivation of the mPFC eliminated this differential intake at the beginning of the session (compare the upper plots in **Figure 7A**). However, the rats did come to lick less for the lower-value solution over the course of the testing session, typically within a few cycles of receiving the two levels of sucrose. This finding is strong evidence against the mPFC having a primarily inhibitory role in the task. The rats could suppress intake of the lower value fluid, but seemed to forget to do so at the beginning of the session. This result suggests that the mPFC is necessary for the expression of the learned feeding strategy, and not the ability to inhibit inappropriate actions. Similar findings on the role of the same mPFC areas (the prelimbic cortex) in the expression of learned fear-based actions have recently been reported (Sierra-Mercado et al., 2006; Lee and Choi, 2012). In these studies, rats fail to express previously learned freezing behaviors at the beginning of the testing sessions but are able to learn to freeze to a stimulus during sessions with mPFC inactivated.

What then is the role of the mPFC in the expression of learned feeding strategies? Insights into this issue were obtained by examining temporal patterns of licking. A “microstructural analysis” (Davis and Smith, 1992) revealed that mPFC inactivation reduced the duration of licking bouts when the higher value fluid was available and increased the number of short-duration licking bouts for lower value fluid (**Figures 3C** and **4B,D**). Previous studies suggest that bout duration is proportional to the absolute concentration of sucrose (or other nutrients) in the fluid, is driven by the orosensory properties (i.e., palatability) of the solutions (Davis, 1973), and depends on processing in the orbitofrontal cortex (Gutierrez et al., 2006). This finding has led to the view that bout durations reflect the animal’s hedonic reaction to an ingested fluid (Dwyer, 2012). However, this interpretation is challenged by studies which show that reductions in reward value, e.g. by reducing fluid volume (Kaplan et al., 2001), can also increase the duration of licking bouts. To address this issue using simple measures, we calculated the ratios of lick counts and bout durations for the task epochs when the higher and lower concentration solutions were available (bottom row in **Figure 3C**). This analysis revealed that the incentive contrast effect was reduced by inactivation of the mPFC. However, as described above, the rats were able to eventually re-implement the suppression of intake of the lower value sucrose solution (**Figure 7**). An interpretation based on incentive contrast effects alone is therefore not sufficient to explain the behavioral effects of mPFC inactivation.

Perhaps the simplest explanation is that the mPFC, especially its rostral most part, is crucial for the maintenance of persistent licking. The brief licking bouts found with mPFC inactivated (**Figure 3B**) are reminiscent of the brief lever presses and head entries that are commonly found in operant tasks following mPFC inactivation, e.g. (Narayanan et al., 2006). How would such effects impact the expression of incentive contrast? With mPFC inactivated, it would not be possible to express the learned feeding strategy, the “rules of the game” (Miller and Cohen, 2001). A simple rule for the incentive contrast task is: Lick persistently when a relatively high level of sucrose is available and suppress licking when a relatively low level of sucrose is available. Perturbing rostral mPFC processing resulted in rats not able to follow this rule. They licked less and emitted briefer bouts when the higher concentration of sucrose was available. They licked a bit more and emitted more bouts when the lower concentration of sucrose was available. Crucially, the effects of rostral mPFC inactivation were not inhibitory: Rats were able to reduce intake of the lower value fluid during the inactivation sessions (**Figure 7**). They simply failed to follow the learned rule for solving the task.

While we found no evidence for a role of the mPFC in inhibitory control over intake, we did find increases in consumption, especially for the lower value fluid, when the ventral striatum was inactivated (**Figure 8**). Together, our studies on the mPFC and ventral striatum suggest that these two brain areas have complementary influences over intake. This effect that may arise through convergent connections from the mPFC and ventral striatum to midbrain centers that are crucial for the control of feeding. For example, tract-tracing studies on the mPFC have reported descending projections from the rostral mPFC to the lateral hypothalamus (Floyd et al., 2001). Other studies on the ventral striatum have reported projections from the ventral striatum to what appears to be this same hypothalamic area (Sano and Yokoi, 2007). No published study has directly compared projections from the mPFC and ventral striatum to these feeding centers in the hypothalamus, e.g., using dual fluorescent tracers. This seems to be a crucial missing piece of the puzzle for understanding how these brain areas contribute to the control of food consumption.

## Author Contributions

Study design: MP and ML; Pilot studies: BL and ML; Data collection: MP and DW; Development of code for data analysis: MP, BL, and ML; Data analysis: MP and LA; Data interpretation: MP, LA, and ML; Writing and Figures: MP, LA, ML. NSF grant 1121147, NIH grant DK099792-01A1, and two grants from the Klarman Family Foundation to ML.

## Conflict of Interest Statement

The authors declare that the research was conducted in the absence of any commercial or financial relationships that could be construed as a potential conflict of interest.

## Footnotes

1 http://www.stanford.edu/group/dlab/cgi-bin/graph/chart.php

2 www.r-project.org
